# Step-By-Step Modeling and Demetallation Experimental Study on the Porous Structure in Zeolites

**DOI:** 10.3390/molecules27238156

**Published:** 2022-11-23

**Authors:** Pavel Kononov, Irina Kononova, Vyacheslav Moshnikov, Evgeniya Maraeva, Olga Trubetskaya

**Affiliations:** 1 Department of Descriptive Geometry and Graphics, Faculty of Basic and Human Sciences, Saint-Petersburg Mining University, 2, 21st Line, 199106 Saint-Petersburg, Russia; 2 Department of Micro- and Nanoelectronics, Faculty of Electronics, Saint-Petersburg Electrotechnical University “LETI”, 5, pr. Popova, 197022 Saint-Petersburg, Russia

**Keywords:** zeolites, computer-aided design, internal structure, pore geometry, nanoarchitectonics

## Abstract

The organization of microporous space in zeolites is discussed. A new step-by-step model is proposed that explains the principles of organizing the hierarchy of microporous space at the stage of assembling zeolites from elements of minimal size: a primary building unit, secondary building units, tertiary building units or building polyhedra, a sodalite cage, and a supercage. To illustrate the stepwise hierarchical porous structure of nanomaterials, the following zeolites with small and large micropores have been selected as the model objects: sodalite (SOD, the maximum diameter of a sphere that can enter the pores is 0.3 nm) and zeolites of type A (LTA, the maximum diameter of a sphere that can enter the pores is 0.41 nm), type X, Y (FAU, the maximum diameter of a sphere that can enter the pores is 0.75 nm), and type BETA (the maximum diameter of a sphere that can enter the pores is 0.67 nm). Two-dimensional and three-dimensional modeling in 3Ds Max software was used. We believe that such an approach will be useful for developing ways to create complex zeolite compositions for specific applications, such as catalysis, where the geometry of the pores determines the size of the molecules entering the voids and computer modeling can play an important predictive role. This work takes a look at specific aspects of using the heat desorption method to study mesoporous materials with a BETA zeolite as an example and presents the results of experimental research into the characteristics of the porous structure of hierarchically structured zeolite materials (specific surface area 180–380 m^2^/g, external surface area 120–200 m^2^/g, micropore volume 0.001–0.1 mL/g).

## 1. Introduction

Materials science has been developing intensively in recent years. A few decades ago, materials physics and chemistry relied on the notion of point defects within a homogeneous area [1], the control of the concentration of which allows one to define the material properties (hardness, concentration of charge carriers in semiconductors, etc.). The following statement was considered a rule of thumb in materials science: “Thermodynamic and kinetic conditions of materials production and processing ensure a given composition within the homogeneous area and the properties of the materials obtained” (from classical thermodynamics, where it was necessary to set the temperature and obtain the material, while now it is required to ensure additional conditions, in particular the component pressures of one of the elements (if it is a binary compound), to stabilize the number of point defects in the crystal structure). When materials science started to explore nanomaterials [2,3,4], this formula evolved into a more complex dependence, as the properties of materials also depend on the size of the nano-object and its shape [5,6,7]. Therefore, it is necessary to introduce the following more complex version of the rule above: “Thermodynamic and kinetic conditions of materials production and processing, as well as the size of nano-objects and their shape, determine properties of the materials [8,9]”. Therefore, issues such as growth control and element orientation in nano-objects have become the main concern of materials scientists [10,11,12]. New trends are appearing in materials science, such as oriented attachment [13,14,15], mesocrystals with mineral bridges, etc. Quasicrystalline concepts of materials and fractality are evolving. In oriented attachment, there are basic elements that can be combined into larger nano-objects. In view of the above, the creation of and development of insights into the formation of nano-objects in the nanoworld is of particular importance for materials scientists. At the same time, such objects as zeolites (porous bodies characterized by a specific skeleton structure and regular geometry of pores, i.e., intracrystalline cavities and channels whose dimensions are comparable with the diameters of molecules) are the most important objects for mastering modern concepts in materials science development.

The primary building units in zeolites are similar to those of mesocrystals but smaller, and regularities in their structure make it possible for the shape to vary, creating unique materials science structures in which a set (a hierarchy) of porous spaces is also formed. Such objects are of great interest for the development of catalysis, photonics, sensing technologies and other modern fields of science and technology, because of the unique opportunities that result from the optimization and control of their functional properties. For example, large pores in porous hierarchical structures can supply materials for a reaction and remove the reaction products, while smaller-sized pores can affect changes in the regions where electron density is exchanged.

Zeolites as model objects are of particular interest because, on the one hand, they are made up of units, and, on the other hand, these units have the smallest sizes, compared to those used in oriented attachment and mesocrystals. They are much required for various methods, so learning the crystallographic understanding of nanoarchitectonics using zeolites as an example is highly useful, for example, when training Master students, so that they can widely introduce these new trends in developing new materials.

The application of zeolite has become well established in various fields, such as the manufacturing of adsorbents and gas separation [16], detergents, the refining and petrochemical industries [17], agriculture and horticulture [18], pollution control, and hydrogen storage. Zeolites are used for industrial, agricultural, and municipal wastewater treatment [19]. Zeolites have been shown to act as anti-cancer agents [20] when mechanically activated through the animal in in-vivo studies and in in-vitro cell models. Zeolites have also found applications in other medical contexts [21].

Zeolites exhibit an unrivaled combination of properties [18], such as large surface area, well-defined microporosity, high temperature resistance, high number of active centers, intrinsic acidity and the ability to retain active metal particles in their pores.

Zeolites are widely used as catalysts and adsorbents in many petrochemical processes. Nowadays, zeolites are widely used as solid catalysts, ion exchangers, and molecular sieves in a number of important industrial processes [19,20]. However, using them for acid catalysis leads to a number of problems, one of the being the high activity and heterogeneity of their acid centers in the given crystal or even the whole sample. Therefore, turning base products into by-products is beneficial. Yet some of these products can be rather bulky and get trapped in micropores thus blocking them [22]. This may lead to the inefficient use of the volume of zeolite crystals and catalyst deactivation. As a result, one of the aims of developing zeolite catalysts is the prevention of the formation of such bulky by-products. This can be achieved through the use of either mesopores or macropores that serve as transport pores [23].

Zeolites offer an unrivaled combination of useful properties, such as a big surface area, pronounced microporosity, high thermal resistance, a large number of active centers, their own acidity, and the ability to trap active particles in their pores. However, their molecular size (less than 2 nm) limits the physical transfer of molecules to and from active centers contained within the micropore network. This has led to scientists’ interest in developing hierarchically structured zeolites. Introducing mesoporosity into an entirely microporous matrix is a suitable method of increasing catalyst efficiency in chemical processes [24,25]. Mesopores provide better access to micropores and reduce diffusion length thus acceleration diffusion and, as a result, catalyst efficiency [26].

It should be noted that zeolites and zeolite-like materials are characterized by well-defined, structurally determined and predictable pore systems, with the pore sizes mainly falling within the range of micropores and usually not exceeding 1.2 nm. At least one additional, usually larger pore system has to be incorporated into the zeolite crystal or added to the crystal structure in order to create a hierarchical [27,28,29,30] pore system with the zeolite pores as the main functional component. The interaction of the two pore systems creates a hierarchy [31].

A distinction is made among zeolites [32,33] based on micropore size, and the groups range from zeolites with small micropores (0.3–0.45 nm, A), through medium (0.55 nm, pentasils) and large (0.75 nm, faujasites, β—0.64 × 0.76 nm) to extra large (>0.8 nm, mordenites).

It is useful to remember that the IUPAC recommendations differentiate three basic types of pores, i.e., micropores, smaller than 2 nm; mesopores, between 2 nm and 50 nm; and macropores, larger than 50 nm. This classification is based on the differences in the basic mechanisms of the sorption processes that occur in pores of different sizes.

In some cases, the porous structure parameters of zeolites play a key role in achieving the required characteristics. For example, the formation of mesoporosity in a microporous zeolite matrix is one of the methods for increasing the efficiency of zeolite materials in catalysis. In earlier studies, work on hierarchically ordered zeolites is mainly devoted to aspects of their synthesis [34], whereas significantly fewer articles are devoted to methods for determining their characteristics. The molecular size of micropores limits the physical transfer of molecules from active centers enclosed in a network of micropores, and this small size significantly limits the number of applicable research methods. In the present work, both the total specific surface area and the external one (excluding the micropores) are studied.

The structures of zeolite surfaces clearly control the access of molecules into the crystals, so computer simulation plays an important predictive role. In this paper, we propose to consider step-by-step the principles of organizing the hierarchy of microporous space at the stage of assembling zeolites from elements of minimal size: a primary building unit, secondary building units, tertiary building units or building polyhedra, a sodalite cage, and a supercage. We believe that such an approach will be useful for the selection and purposeful development of hierarchical zeolites for an intended specific application, for example, in catalysis, where the geometry of the pores determines the size of the molecules entering the voids.

Zeolites and zeolite-like materials are characterized by clearly defined and structurally determined and predictable pore systems; the pore size is typical of that of micropores and does not exceed 1.2 nm. To create a hierarchical pore system [35,36,37] whose functional component is zeolite pores, at least one additional (usually bigger) pore system must be imbedded in the zeolite crystal or the crystal structure. The interaction between the two systems creates a hierarchy.

In earlier works studying hierarchically structured zeolites, zeolite synthesis has been the main focus, while fewer papers have looked at the methods used to determine their characteristics. Nowadays, a lot of research is being conducted using the BET method, but researchers understand the need to be careful when using this method to analyze the surface area of hierarchical zeolites, since the presence of micropores renders the BET theory inaccurate. In this work, both the full specific surface area (S_BET_) and the outer surface (excluding micropores—STSA) are studied. The choice of the research method depends on the partial pressure of the adsorbent gas [36].

The available literature does not describe a step-by-step model of the organization of the hierarchy of the microporous spaces of zeolites, starting from the primary building blocks of elements of minimum size and ending with the elementary cell of zeolite. The objective of this work, therefore, was to study the nanoarchitectonics of forming porous spaces in nanomaterials made up of small primary units that form a hierarchy of the microporous space (with small and large micropores); to do so using the example of a clear, step-by-step creation of structure in sodalites (SOD) and zeolites of type A, type FAU, and type BETA using 2D and 3D modeling; and to find the specific features of the sorption analysis method used to study the influence of chemical postsynthetic processing on the characteristics of the porous structure of hierarchical zeolites using a Sorbi device.

## 2. Results

### 2.1. Principles of Organizing the Hierarchy of Microporous Space at the Stage of Assembling Zeolites from Elements of Minimal Size

**Figure 1:**
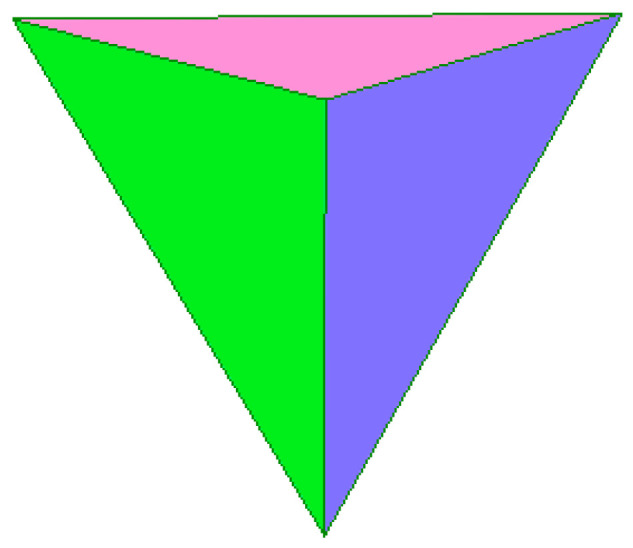
A primary zeolite building unit (the first stage in modeling the three-dimensional structure of zeolites) [compiled by the authors].

**Figure 2:**
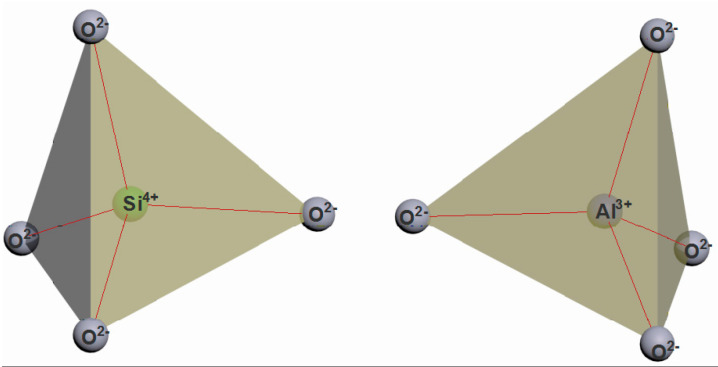
TO_4_ tetrahedra [compiled by the authors].

The construction of the zeolite cages starts with combining the two primary building units (PBU) of zeolites, i.e., the tetrahedra (Figure 1) TO_4_ (where T are silicon or aluminum atoms), into a regular polyhedron whose faces are four equilateral triangles. The vertices of the tetrahedron are occupied by silicon and aluminum atoms (Figure 2, the average bond length between silicon and oxygen is 0.164 nm, the O-O distance is 0.263 nm, the Si-Si distance is 0.310 nm). The O-T-O bond angle is slightly (±2–3°) different from the tetrahedral one (109°28′).

In zeolites, aluminum, as well as silicon, is in tetrahedral oxygen coordination and isomorphically replaces silicon in the silicon-aluminum-oxygen cage. The silicon-to-aluminum ratio in a zeolite lattice may vary within quite a wide range, but there is an upper limit for aluminum. Loewenstein [38] formulated the rule of thumb, stating that the aluminum content in the tetrahedral positions in silica-alumina cages can be either less than or equal to the silicon content, but cannot exceed it. This rule implies that only Si-O-Si and Si-O-Al bonds are possible in zeolite cages, whereas Al-O-Al bonds are not possible. Therefore, the Si-to-Al ratio cannot be less than 1.

**Figure 3:**
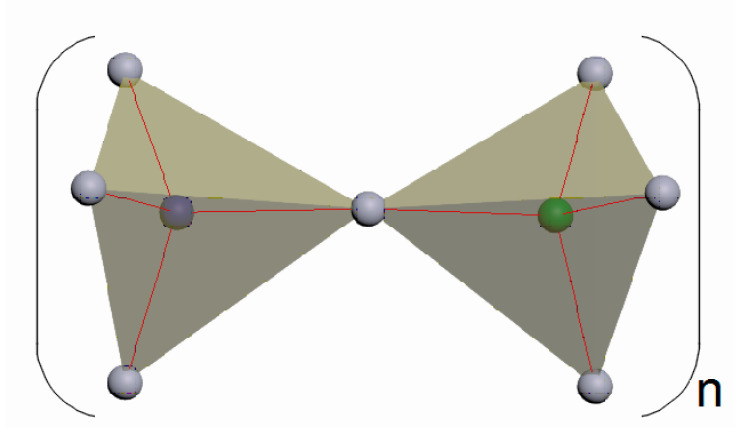
Bonding of TO_4_ tetrahedra via oxygen atoms (n is the number of Si-O bonds) [compiled by the authors].

The alternating silica-oxygen and alumina-oxygen tetrahedra combine (the association is made by the aggregation of only the tetrahedron vertices, not their edges or faces) into three-dimensional zeolite cage structures so that all the oxygen atoms are divided between the two adjacent tetrahedra (Figure 3).

Thus, the second stage of modeling the self-assembly of zeolite structures involves the bonding of the tetrahedra (the T-O-T angle between the tetrahedra varies around 140°) via the oxygen atoms as well as the formation of secondary building units (SBU) and tertiary building units (TBU), or building polyhedra.

**Figure 4:**
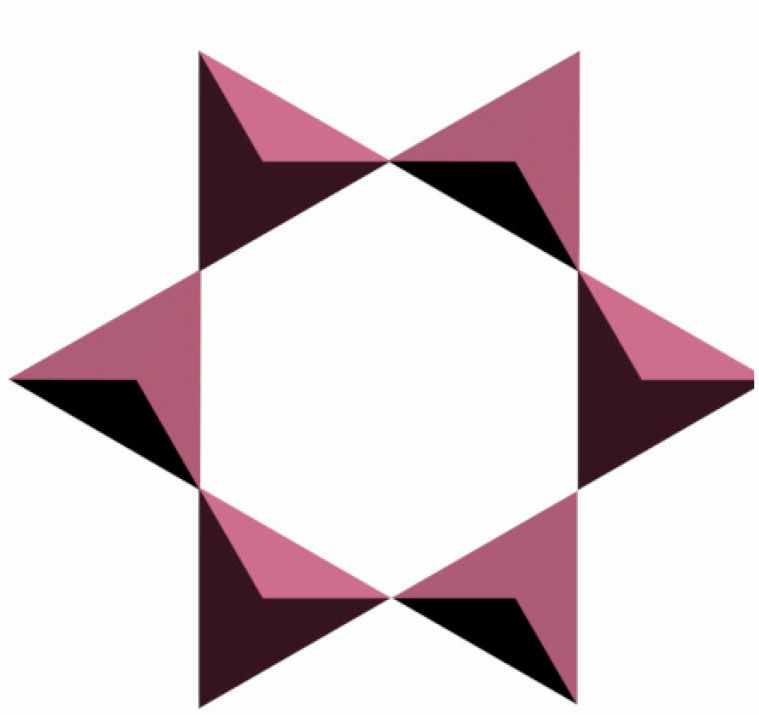
A six-membered ring made of TO_4_-tetrahedra (secondary building unit) [compiled by the authors].

The secondary building units represent four-, five-, six-, eight-, ten-, and twelve-membered rings of TO_4_-tetrahedra. Figure 4 shows a six-membered ring of six tetrahedra as an illustrative example.

The tertiary building units are prisms and more complex polyhedra.

**Figure 5:**
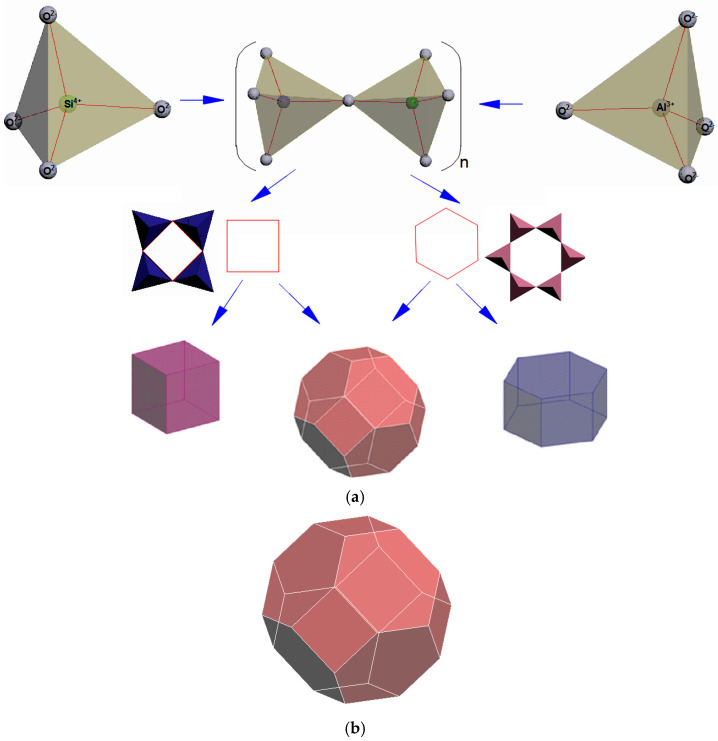
(**a**) Combination of four-membered and six-membered rings into a sodalite cage; (**b**) A polyhedron, i.e., a truncated octahedron, is the so-called sodalite cage (*β cage* (*SOD*)), formed by 24 tetrahedra (a tertiary building unit) [compiled by the authors].

**Figure 6:**
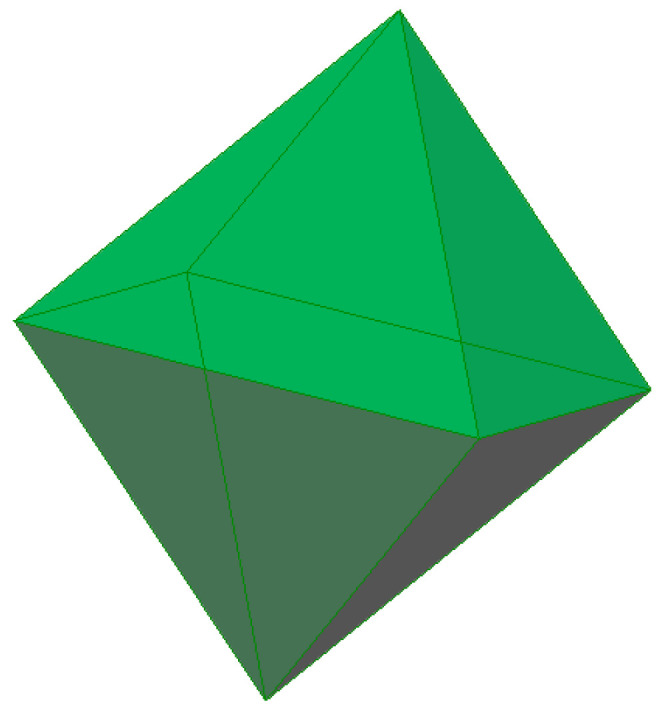
A convex regular octahedron [compiled by the authors].

The four-membered and six-membered rings combine to form a tertiary structural unit in the form of a polyhedron, a truncated octahedron called a sodalite cage, or β cage, formed by 24 tetrahedra (Figure 5). It should be noted that the convex regular octahedron (Figure 6) is a polyhedron with 8 faces, all which are equilateral triangles, and the truncated octahedron is a polyhedron with 14 faces, 6 of which are squares and 8 of which are regular hexagons. Two hexagonal faces and one square face converge at each of its twenty-four identical vertices (Figure 5). There are 24 T-atoms at the vertices of the polyhedron, with the oxygen atoms located between them.

Its free diameter β cage is 0.66 nm, and its point symmetry is m3m.

**Figure 7:**
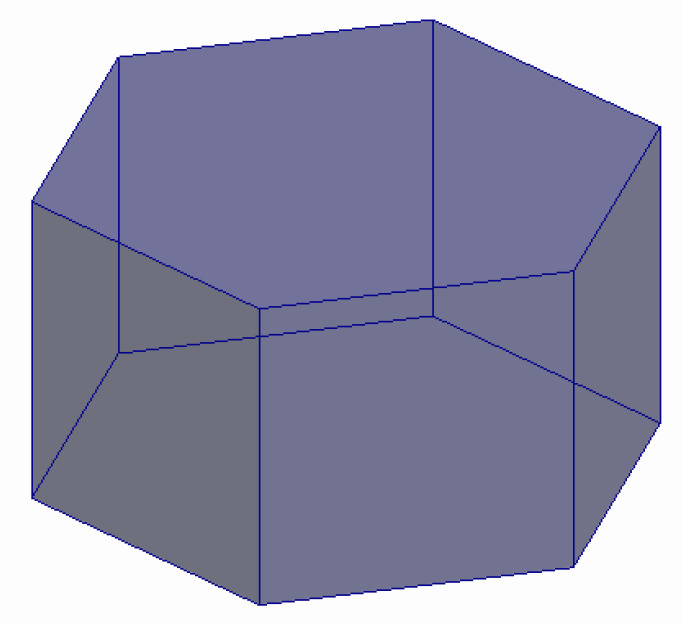
A hexagonal prism (D6R) consisting of 2 six-membered and 6 four-membered rings made up of TO_4_-tetrahedra (a tertiary building unit) [compiled by the authors].

**Figure 8:**
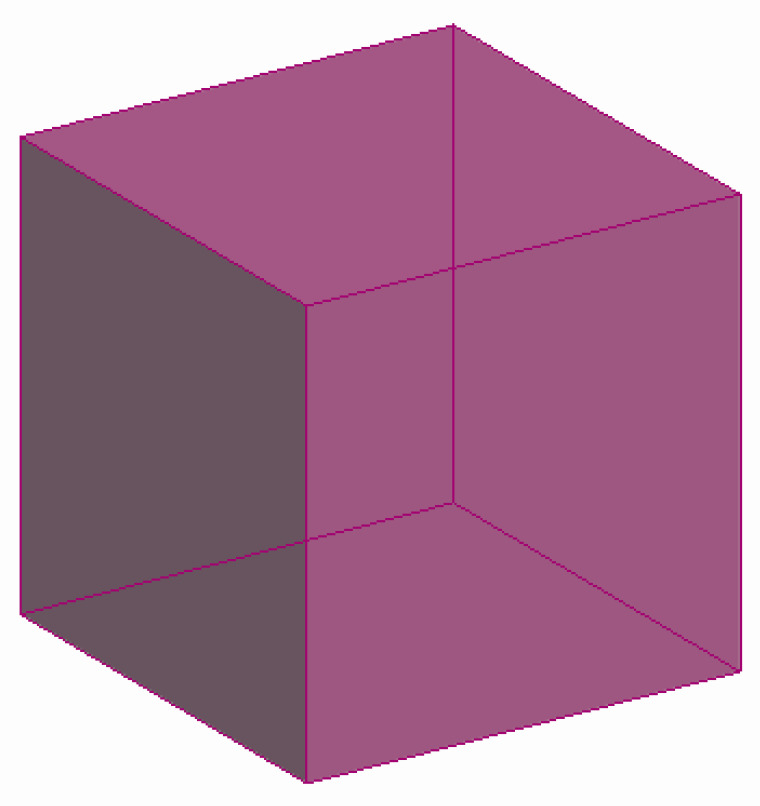
A cube (D4R), consisting of 6 four-membered rings (a tertiary building unit) [compiled by the authors].

Figure 7 shows a hexagonal prism (D6R) consisting of 2 six-membered and 6 four-membered rings, while Figure 8 illustrates a regular quadrangular prism (D4R, i.e., a cube) consisting of 6 four-membered rings.

Specific arrangements of the secondary (SBU) and tertiary (TBU) building units of zeolites lead to significant differences in the type and morphology of different zeolite varieties.

This paper describes specific structural features on the example of small pore zeolites, using sodalite (SOD) and zeolites of type A (LTA—Linde Type A), as well as large pore zeolites, using type FAU (zeolites X and Y) and type BETA as examples. The latter type of zeolites are structural analogues of faujasite, the difference between the X and Y cages being the Si-to-Al ratio, which is from 1.0 to 1.5 for type X zeolites and from 1.5 to 3 for type Y zeolites. Connecting the truncated octahedron with the four-membered prisms (with the cavity diameter of 0.23 nm) made up of T-atoms produces type A zeolites, while doing the same with the six-membered ones (0.36 nm) yields type X and type Y zeolites.

A unit cell is formed during the third modeling stage.

**Figure 9:**
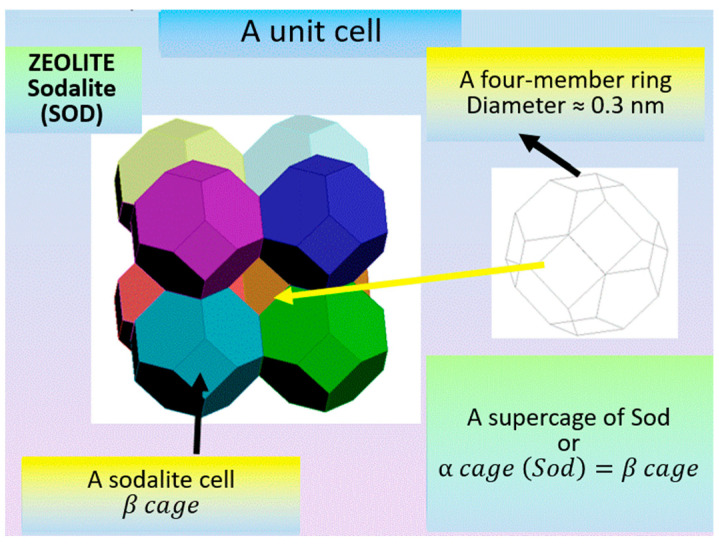
Sodalite unit cell [compiled by the authors].

Figure 9 shows a unit cell of a sodalite zeolite that includes a sodalite cell or β cage, which is a truncated octahedron and is formed by 24 TO_4_-tetrahedra.

**Figure 10:**
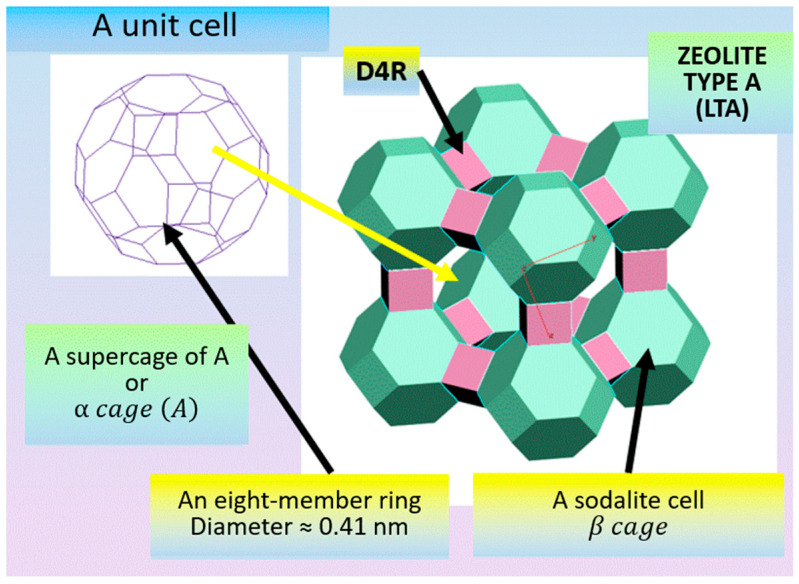
Type A sodalite unit cell [compiled by the authors].

A Type A sodalite unit cell (Figure 10) includes:

- a sodalite cell or β cage, which is a truncated octahedron and is formed by 24 TO_4_-tetrahedra;
- cubes, consisting of 6 four-membered rings;
- a supercage A or α cage (A), the diameter of whose eight-membered ring is approx. 0.41–0.43 nm.

Cubic units (paired quadruple rings), located at the midpoints of the unit cell edges, produce, when joined, cavities at the vertices of the β cage cubic unit cell which have the shape of a truncated octahedron. The 12 lateral faces of the cubic units in the center of the unit cell form a large cavity (supercage of A or α cage (A)) that has the shape of a semiregular polyhedron, a truncated cuboctahedron (with 12 square faces, 8 regular hexagon faces, and 6 regular octahedron faces) with the point symmetry of m3m. The free diameter of the α cage (A) is 1.14 nm, and it communicates with the eight adjacent β cage through the six-membered windows with the diameter of about 0.22 nm. α cage (A) is connected besides the β cage with six other α cages (A) by eight-membered windows with diameter of around 0.41–0.43 nm. This creates a system of intersecting end-to-end channels in the direction of {100}. The centers of the α cage (A) form a primitive cubic lattice.

**Figure 11:**
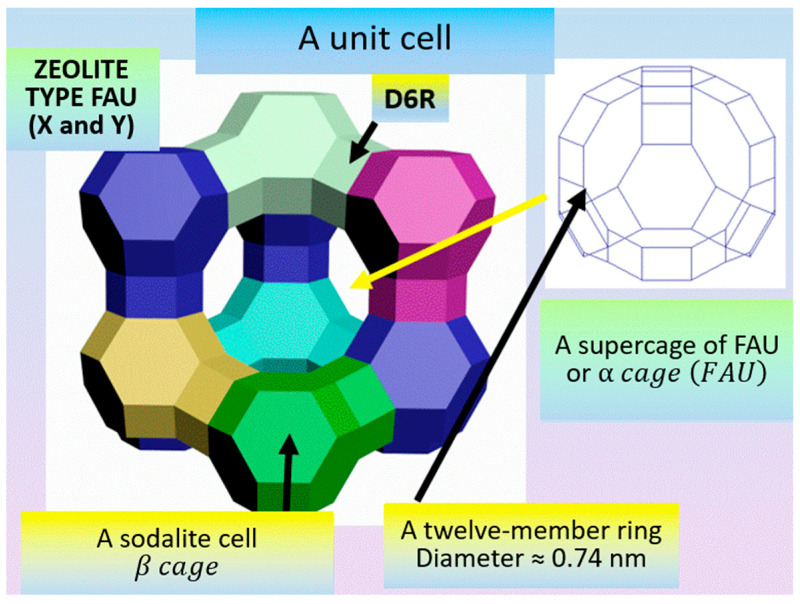
Type FAU sodalite unit cell [compiled by the authors].

The type FAU sodalite unit cell (Figure 11) includes:

- a sodalite cell or β cage, which is a truncated octahedron and is formed by 24 TO_4_-tetrahedra;
- hexagonal prisms that consist of 2 six-membered and 6 four-membered rings;
- a supercage FAU or α cage (FAU)), the diameter of whose eight-membered ring is 0.74 nm.

The aluminosilicate cage of FAU is made up of paired six-membered rings. By joining with their vertices, four such rings form a β cage; however, the point group of symmetry of the sodalite cell is lowered to the group of $\bar{4}3\;m$ on account of its environment. When further construction is continued, a large-size cavity with the diameter of $\sim 1.2\;{}_{HM}$ and the point symmetry of $\bar{4}3\;m$ is formed between the 10 sodalite cells, with 4 twelve-membered windows leading to this cavity. When the large-size cavities are joined together, a system of six intersecting zigzag channels is created in the direction of {110}. The centers of the large cavities form a lattice with Fd3m symmetry in the space.

**Figure 12:**
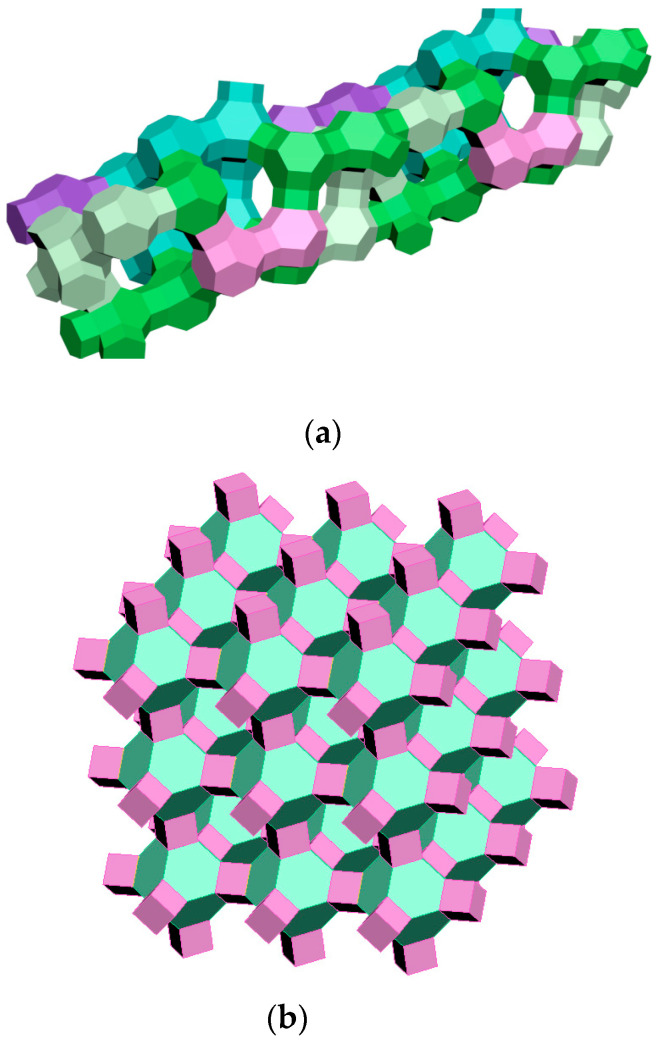
Three-dimensional structure of zeolites: (**a**) FAU type zeolite (X and Y); (**b**) A type zeolite (LTA) [compiled by the authors].

The fourth stage of modeling the self-assembly of zeolite structures, which illustrates the three-dimensional structure of zeolites, as well as the FAU type and A type, is shown in Figure 12.

**Figure 13:**
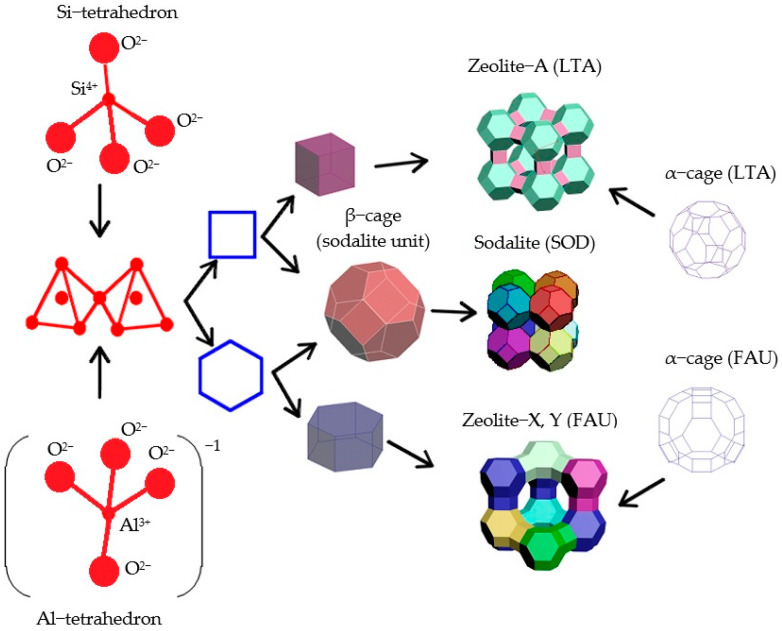
Hierarchical diagram of the shaping of the porous structure of zeolites [compiled by the authors].

Figure 13 shows a hierarchical diagram of the shaping of the porous structures of the zeolites in this study.

**Figure 14:**
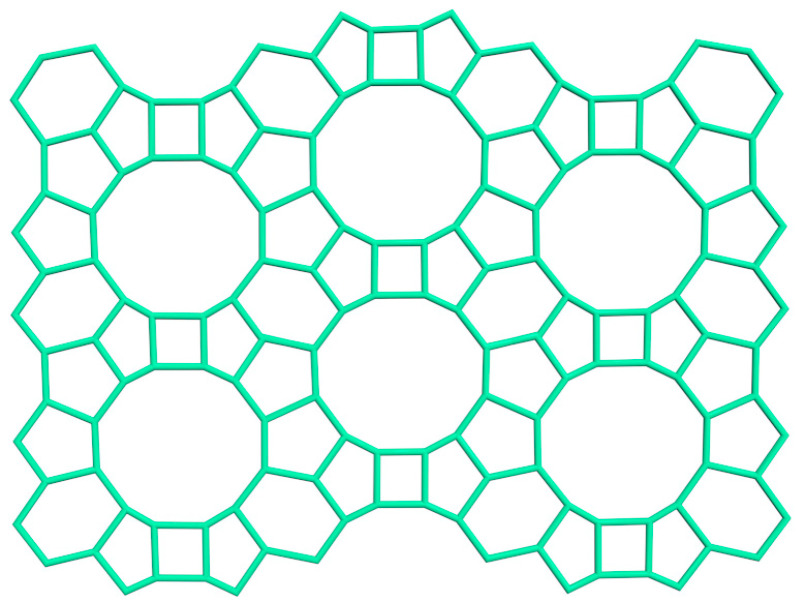
The structure of the zeolite type BETA [compiled by the authors].

**Figure 15:**
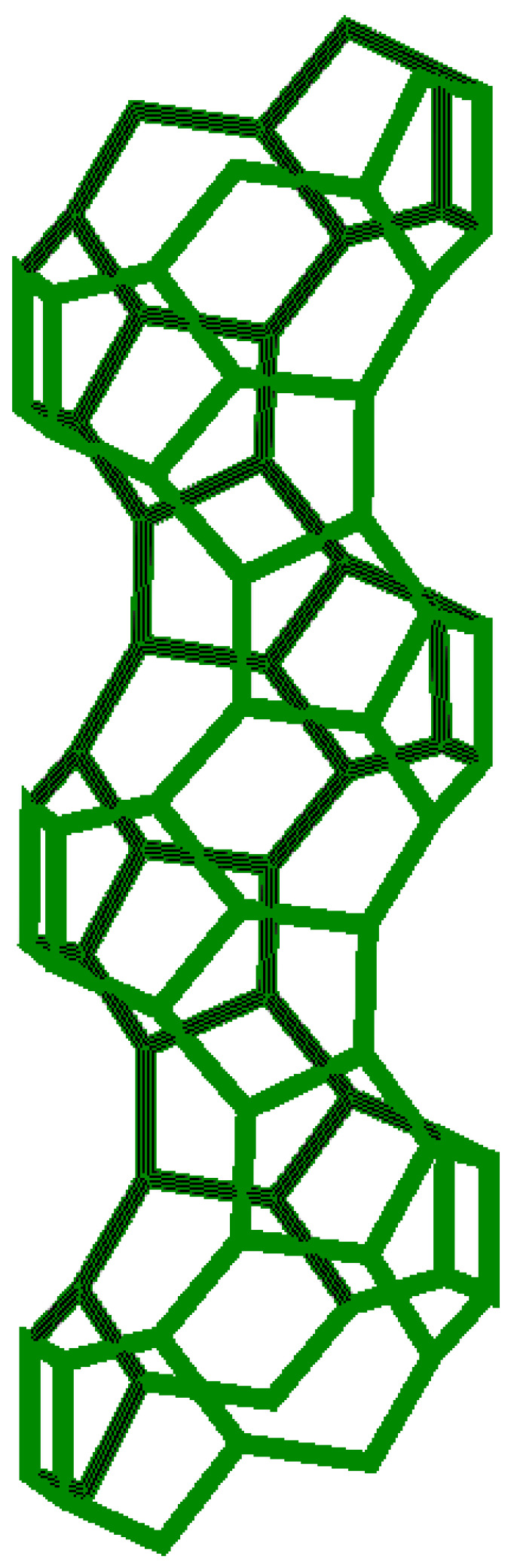
12-ring channel of the zeolite type BETA [compiled by the authors].

**Figure 16:**
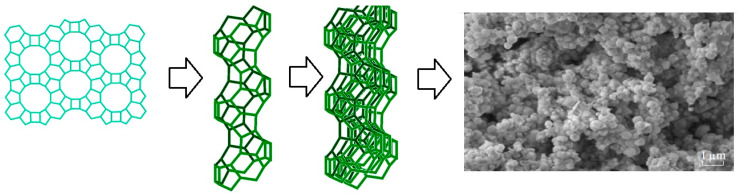
Design for zeolite synthesis type BETA [compiled by the authors].

Figure 14, Figure 15 and Figure 16 represent the structure of the BETA type zeolite (the maximum diameter of the sphere that can enter the pores is 0.67 nm, the maximum diameter of the sphere that can pass through is 0.59 nm).

It is obvious that, with the partial removal of silicon or aluminum atoms, the diameter of the sphere will increase.

### 2.2. Investigation of Hierarchically Organized BETA Type Zeolites by Sorption Analysis

The aim of the TDM experiment was to identify micropores and measure the specific surface area, and that is why a range of relative partial pressures of the adsorbent gas P/P_0_ of up to 40% was chosen. As a result of the analysis of the adsorption isotherms, data related to the specific area size and micropore size were gathered. The results of the characteristics of the porous structure of the samples produced during the experiment are shown in Table 1.

Samples 2,3 were treated with solutions of HCl of different concentrations to dealuminate them. Table 1 shows that, when zeolite 2 M is treated with a solution of HCl (sample 2), its specific surface area increases to 384 m^2^/g, but the volume of its micropores decreases slightly, from 0.104 m^2^/g to 0.097 m^2^/g, while treating it with a 0.1 M solution of HCl leads to a decrease in both its size and the volume of its micropores.

Samples 4,5 were treated with solutions of NaOH of different concentrations to recrystallize and desilicate them. When treated with a 2 M solution of NaOH (sample 4), both the specific surface area and the volume of the micropores decrease significantly—to 179 m^2^/g and almost by a factor of one hundred, respectively. When treated with a 0.1 M solution of NaOH (sample 5), its specific surface area decreases to 269 m^2^/g and the volume of its micropores to 0.035 cm^2^/g.

**Figure 17:**
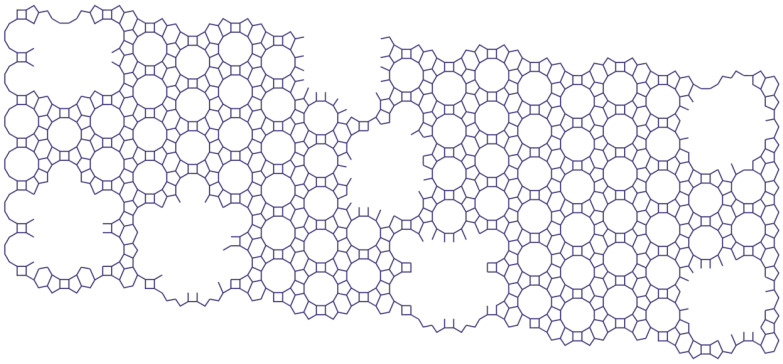
The model structure of sample 2 [compiled by the authors].

**Figure 18:**
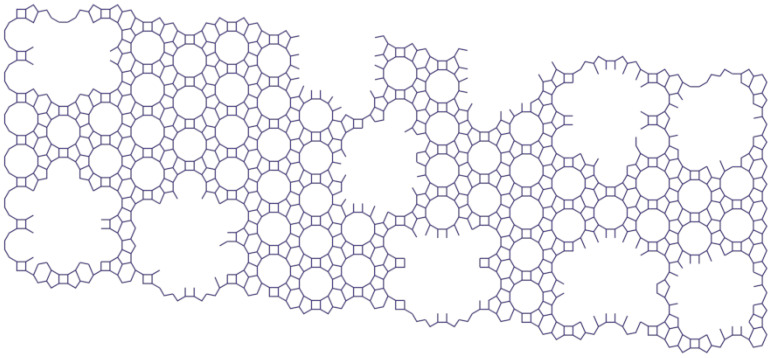
The model structure of sample 3 [compiled by the authors].

The results of the micropore volume, the total pore volume, and the percentage ratio of micropore volume to total pore volume are shown in Table 1. After treatment with a solution of 2 M HCl, the percentage ratio of micropore volume to total pore volume decreased by 20.4% compared to the original sample (Figure 17), and, after treatment with a solution of 0.1 M HCl, by 27.5% compared to the original sample (Figure 18). The mesopores larger than 2 nm came into being when the -Si-O- and -Al-O- bonds broke and micropores combined.

**Figure 19:**
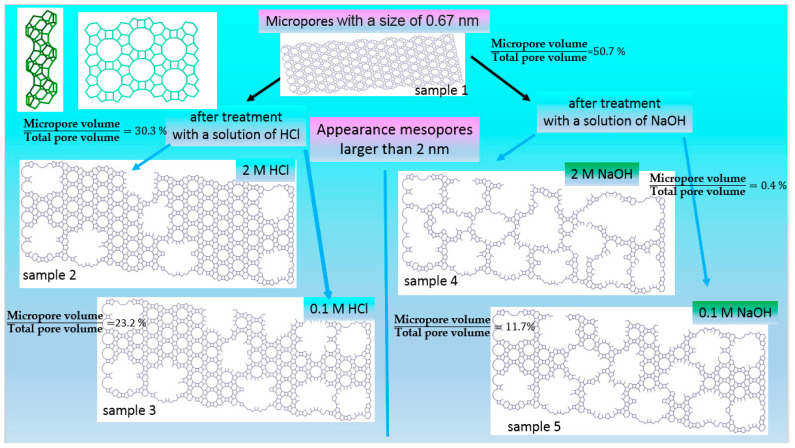
The model structures of samples [compiled by the authors].

Figure 19 shows the model structures of samples 1–5.

## 3. Conclusions

In this work, three-dimensional modeling in the 3ds Max environment was applied to the study of the regularities of the processes in the nanoworld, namely, the study of the nanoarchitectonics of the formation of porous spaces on the example of a step-by-step model of the construction of the zeolite structure. Zeolites as a model variant were especially interesting, since, on the one hand, they consist of bricks, and, on the other hand, these bricks are the smallest (compared to those used in oriented attachment and in mesocrystals).

This work takes a look at specific aspects of using the heat desorption method to study mesoporous materials with a BETA zeolite as an example, and presents the results of experimental research into the characteristics of the porous structure of hierarchically structured zeolite materials. The specific surface area and volume of micropores have been established. After dealumination and desilication of zeolites, the volume of micropores decreases in both cases, but, in case of desilication, both the specific surface area and the volume of micropores decrease more significantly. This is consistent with the results of computer modeling (with partial removal of silicon or aluminum atoms, the diameter of the sphere will increase). Acid treatment leads to the removal of extraframework particles and the formation of mesopores [39]. Very often, such an acid treatment is used to remove amorphous fragments from the zeolite.

Compared to classical synthesis technologies, desilylation and dealumination strategies are cheap, simple, and efficient [34]. Despite great progress in the development of the synthesis of zeolites, there are still many problems that need to be solved. For example, although many zeolites can be easily synthesized in the laboratory, commercial production of zeolite materials is limited.

The development of hierarchical zeolites is a booming area of research [35,40,41,42,43,44,45]. However, in many cases, a more thorough analysis of the relationship between hierarchization and possible improvement in properties is still needed [36]. Moreover, hierarchization necessarily creates a large surface without micropores.

Considerable efforts need to be made to transfer the results of scientific research to industrially viable production processes.

From the point of view of studying the geometry of pores, the most attractive prospects open up in the study of a catalytic reaction, when it is possible to predict the details of catalytic processes from the size of voids at the atomic level, such as, for example, the size of the molecules entering the voids. In this case, the proposed model will be predictive.

The proposed step-by-step nanoarchitectonic model of zeolite formation can find potential application in the study of zeolite catalysts. For example, it is well known that the binder has a profound effect on the efficiency of hierarchical zeolite catalysts, either blocking pores or preventing access to active sites, and thereby reducing the performance of catalysts. We believe that the approach of step-by-step consideration of the self-assembly of zeolites of various types and knowledge of the principles of the organization of porous space at the initial level will in the future allow us to develop recommendations on the development of ways to create complex compositions of zeolites and methods of their processing for specific applications.

Scientific novelty is determined by the proposed step-by-step nanoarchitectonic model of zeolite formation, which can be useful for:

- mastering and understanding the crystallographic concept of nanoarchitectonics.
- development and creation by materials scientists of new objects with new properties in the conditions of atomic and molecular design and nanoarchitectonics.
- introducing new directions in the development of new nanomaterials, as well as creating interdisciplinary links among subjects such as “Nanomaterial science”, “Nanotechnology”, and “Descriptive geometry, engineering and computer graphics” in order to develop students’ spatial representation and geometrical way of thinking. Using geometrical simulation to construct zeolite structures and models can actively promote the idea of cross-disciplinary interaction, which in many ways forms the basis of modern educational standards and is in line with the strategic directions of contemporary scientific research.

## 4. Materials and Methods

Zeolites with small and large micropores, i.e., sodalite (SOD) and zeolites type A and type FAU, were selected as the materials. The modeling process in the 3ds Max software suite was conditionally divided into the following stages: setting up units of measurement; building a regular polyhedron, i.e., a tetrahedron, as well as semiregular polyhedra, i.e., a truncated octahedron and a cuboctahedron. The measuring units were set in the following way: in the ‘Display Unit Scale’ section of the ‘Units Setup’ dialog box, the ‘Metric’ option was checked and ‘Millimeters’ were selected from the corresponding drop-down list; then, in the ‘System Unit Setup’ dialog box, the following settings were made: 1 Unit = 1.0 Mm. Modeling of the octahedron in 3ds Max was performed using the ‘Hedra’ primitive from the ‘Extended Primitives’ list. The ‘Cube/Octa’ parameters were set in the ‘Family’ section of the ‘Modify’ tab, and ‘P’—1.0; ‘Q’—0.0 in the ‘Family Parameters’ section. An Archimedean polyhedron, i.e., a truncated octahedron, was obtained by applying the ‘Edit Poly’ modifier, activating the ‘Edge’ level, and selecting all the edges of the object. In the ‘Number of Vertices’ field of the ‘Edges’ panel, the value of two was entered, and the ‘Insert Vertices’ command was executed. All new vertices forming bases of the small pyramids were connected with one another using the ‘Connect’ command, while the remaining vertices of the square pyramids were removed using the ‘Remove’ command.

In the experimental part, this work studies a number of samples of BEA 1–5 zeolites differing in how they are synthesized. Out of the many methods used to synthesize hierarchical zeolite, postsynthetic methods have been chosen, namely dealumination and desilication. These methods are among the most widely used modification tools. They have proved to be simple and efficient. The BEA zeolites were synthesized at Irkutsk National Research Technical University [37]. Table 2 below describes the zeolites used.

The characteristics of the hierarchical zeolites were studied using a gas-adsorbent Sorbi-series analyzer (Meta, CJSC, Novosibirsk, Russia) by comparing the volumes of the adsorbent gas (nitrogen) adsorbed by the sample with the sample (specific surface area 106 m^2^/g) provided by Meta, CJSC.

**Figure 20:**
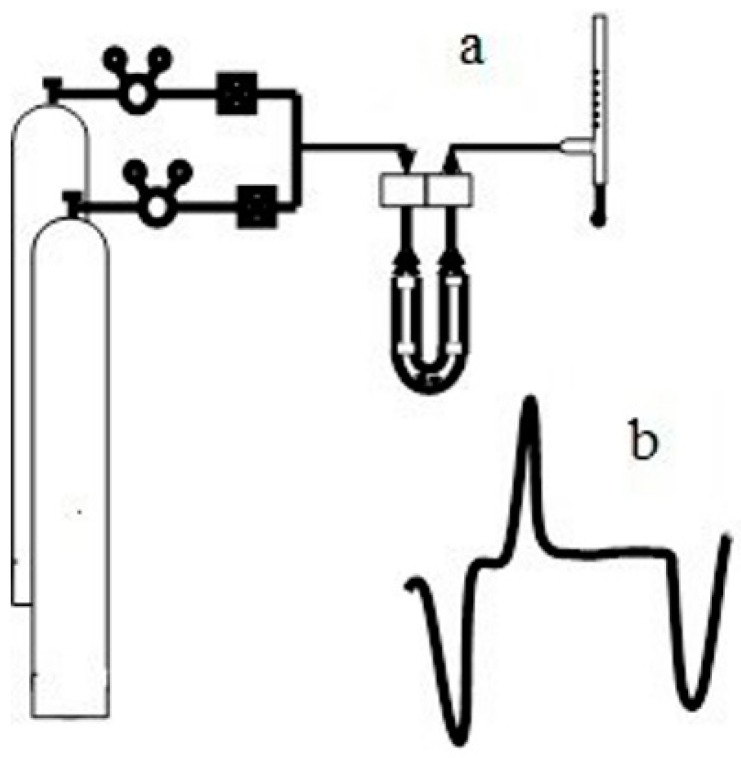
Diagram of plant used to measure specific surface area using heat desorption method (**a**) and adsorption and desorption spikes corresponding to heating and cooling of samples (**b**).

As a rule, the plant compatible with the thermodesorption method (TDM) consists of a gas-preparation part (usually tanks filled with N_2_ or Ar—adsorbent gases, and He—carrier gas), an adsorber—a U-shaped tube, and a gas meter (Figure 20).

Adsorbent training happens inside the adsorber, which is placed inside a furnace at the temperature of 200–350 °C with a He or an Ar/He mixture flowing through it. After that, the adsorber is cooled to room temperature with the gas still flowing (at such a temperature Ar and He normally do not get adsorbed); then the adsorber is placed inside a cryogenic storage dewar filled with liquid nitrogen—at this temperature Ar is adsorbed but the carrier gas (He) still practically does not get adsorbed). After equilibrium saturation at a fixed concentration of Ar, the adsorber is heated to room temperature, the Ar adsorbed at 77 K is released, and its quantity is measure by the thermal conductivity detector (katharometer, META, Novosibirsk, Russia)

The TDM has become most widely used as an express ‘single-point’ method to measure specific surface area. The surface size is then measured using the BEA method, in which, for the purpose of simplification, C is made a constant (C = 60 for Ar adsorption and C = 100 for N_2_ adsorption), or using the comparative method by finding the proportionality coefficient between the surface size and absorption value under fixed conditions. This coefficient can be conveniently found using a standard sample of a known specific surface area.

**Figure 21:**
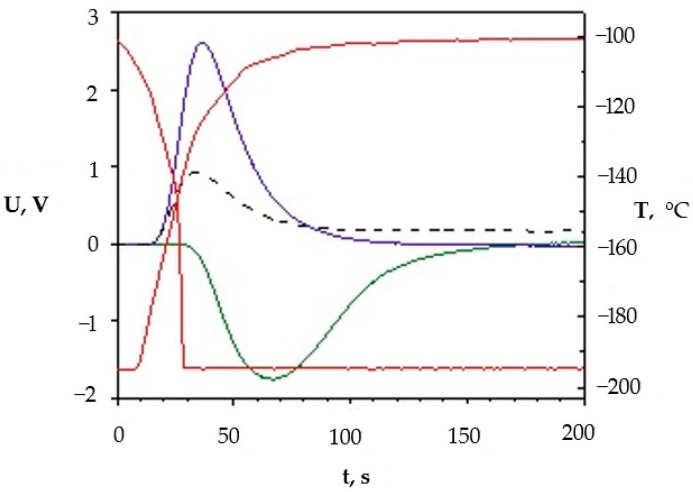
Signals received from thermal conductivity meter formed during adsorption analysis (the red lines-temperature changes; the green line-adsorption signal; the blue line-desorption signal; the black dashed line-changes in the adsorbent gas pressure).

Figure 21 shows signals formed when adsorption–desorption processes are studied using the Sorbi device.

In the figure above, the red lines represent temperature changes. At the first stage, a sample of porous silica is cooled through natural heat transfer between the sample and the medium (liquid nitrogen) until the temperature drops to 77 K (the lower red line). During this process, the inert adsorbent gas is adsorbed, which leads to a change in the adsorption signal (the green line). An increase in the nitrogen concentration in the mixture leads to a change in its thermal conductivity, since (λN2/λHe ~ 1/7).

After the absorption is complete, the ampule containing the sample is removed from the cryogenic storage and heats by approximately 100 K (the upper red line). At the same time, the desorption process begins, accompanied by a desorption spike (the blue line) which has a more defined time. All subsequent calculations are based on the analysis of the size of the graph under the desorption curve. The black dashed line reflects changes in the adsorbent gas pressure when the pressure is set before each cycle of the measurement.

Despite a number of downsides, such as the necessity of using models, the errors inherent in the BET method, and the labor-consuming nature of the analysis (getting a full isotherm can take as long as 8 h), the TDM is a unique method capable of analyzing very small pores (less than 2 nm in diameter, called micropores).

## Figures and Tables

**Table 1:** Results of parameters of porous structure of samples.

Sample Number	1	2	3	4	5
Specific surface area, m^2^/g	358	384	287	179	269
External surface area, m^2^/g	134	171	119	177	196
Micropore volume, mL/g	0.104	0.097	0.079	0.001	0.035
Total pore volume, mL/g	0.205	0.320	0.341	0.265	0.300
Micropore volume/Total pore volume, %	50.7	30.3	23.2	0.4	11.7

**Table 2:** Description of studied samples.

Sample Number	Sample Composition	Comments	Tempering Conditions
1	BEA—original	No treatment	550 °C
2	BEA + 2 M HCl	Treatment method: dealumination. The sample was treated with a solution of HCl at the temperature of 100 °C and continuously stirred for 1 h.	500 °C
3	BEA + 0.1 M HCl	Treatment method: dealumination. The sample was treated with a solution of HCl at the temperature of 100 °C and continuously stirred for 1 h.	500 °C
4	BEA + 2 M NaOH	Treatment method: desilication. The sample was treated with a solution of NaOH to cause recrystallization to induce the formation of secondary mesoporosity.	500 °C
5	BEA + 0.1 M NaOH	Treatment method: desilication. The sample was treated with a solution of NaOH to cause recrystallization to induce the formation of secondary mesoporosity.	500 °C
